# Peripheral vasoreactivity in acute ischemic stroke with hemiplegia

**DOI:** 10.1038/s41598-021-88050-9

**Published:** 2021-04-20

**Authors:** Su Jeong Wang, Chan-Hyuk Lee, Hyun Goo Kang, Ko Woon Kim, Minjoo Kim, Hwan-Jeong Jeong, Byoung-Soo Shin

**Affiliations:** 1 Department of Neurology, Jeonbuk National University Medical School and Hospital, 20 Geonji-ro, Deokjin-gu, Jeonju, 54907 Republic of Korea; 2 Research Institute of Clinical Medicine of Jeonbuk National University-Biomedical Research Institute of Jeonbuk National University Hospital, Jeonju, Republic of Korea; 3 Department of Nuclear Medicine, Jeonbuk National University Medical School and Hospital, 20 Geonji-ro, Deokjin-gu, Jeonju, 54907 Republic of Korea

**Keywords:** Medical research, Neurology

## Abstract

The association between vasomotor tone of the peripheral arteries and cerebral hemisphere function has not been established. This study analyzed the peripheral vasoreactivity of patients with acute ischemic stroke and hemiplegia using a modified Raynaud scan, which is a new technology for blood flow measurement. In this retrospective case–control study, we examined patients with unilateral weakness consistent with ischemic lesions who underwent brain magnetic resonance imaging and modified Raynaud scanning within five days from the onset of symptoms. The modified Raynaud scan was used to quantify the radioactivity of the bilateral fingertips during rest and cooling-heating thermal stress conditions and estimate vasoreactivity based on the change in the blood amount per time under rest-thermal stress. The subjects were classified into the preserved and impaired groups based on their degrees of vasomotor reaction. Based on the modified Raynaud scanning, 37 (mean age = 69.1 ± 10.6) and 32 (mean age = 62.6 ± 11.8) subjects were allocated to the preserved and impaired groups, respectively. Binary logistic regression showed that the affected limb edema (odds ratio (OR) 6.15; confidence interval (CI) 1.40–26.97; *p* = 0.016) and anterior circulation (OR 3.68; CI 1.01–13.48; *p* = 0.049) were associated with impaired vasoreactivity. The modified Raynaud scans confirmed that central lesions in the anterior circulation with hemiparesis may influence the vasoreactivity of edematous peripheral arteries. These results may inform treatment and rehabilitation for stroke patients with hemiparesis.

## Introduction

Vasoreactivity is indispensable for maintaining homeostasis in the human body, as it maintains blood pressure and body temperature within normal limits via the constriction or relaxation of blood vessels. The dysfunction of vasoreactivity can cause several complications, including Raynaud's phenomenon (RP), which is the excessive vasoconstriction of the peripheral arteries in the limbs (mainly fingers) exposed to low external temperatures^1^.

The pathophysiological mechanism of peripheral vasoreactivity at the level of the peripheral nervous system is well-known. It is regulated by the sympathetic nervous system and branched from the T1-L2 segments of the spinal cord. It was confirmed in an animal model that the anterior cingulate cortex and hypothalamus, a portion of the limbic system, are involved at a level higher than the spinal cord^2^. We hypothesized that the cerebral hemisphere, especially the motor cortex, may be involved in vasomotor tone control; however, this hypothesis has not yet been tested.

Laser, Doppler ultrasound, and infrared methods are representative tools for objectively assessing blood flow in the peripheral arteries. These methods indirectly estimate blood flow by evaluating the dispersion of red blood cells (RBCs) or skin temperature^3–5^. Conventionally, the Raynaud scan involves the immersion of a body part in cold water to induce stress, and the results are estimated visually. Here, we designed a new technique, called the ‘modified Raynaud scan,’ involving the use of a thermal stress device to provide cyclic cooling and heating thermal stress that ranged from 15 to 41 °C during stress image acquisition, followed by the analysis of the image data to quantitatively measure the change in blood flow. This study aimed to evaluate the peripheral vasoreactivity of ischemic stroke patients with hemiplegia using this modified Raynaud scan. We also examined the factors associated with peripheral vasoreactivity in ischemic stroke patients.

## Methods

### Patient selection

This was a retrospective case–control study that targeted patients (≥ 18 years old) who were hospitalized due to acute ischemic stroke (within 7 days from onset) from August 2017 to March 2019. As a first step, patients who showed objective unilateral weakness neurologically associated with acute ischemic lesions were selected. Of them, only patients (final subjects) who underwent brain magnetic resonance imaging (MRI) and our modified Raynaud scan within five days from the onset of symptoms were included in the final analysis. The vascular reactivity of both hands was measured using Tc-99 m labeled RBCs. The subjects were classified into two groups (preserved or impaired) according to the degree of reactivity.

The Institutional Review Board of Jeonbuk National University Hospital, Jeonju, Soputh Korea, approved this study (Reference number: CHU 2019-04-028). All procedures were carried out following the ethical standards of the Helsinki Declaration and the institutional and national research committees. After obtaining ethical approval from the Institutional Review Board of Jeonbuk National University Hospital, informed consent was waived due to the retrospective nature of the study.

### Data acquisition

This study examined the demographic factors, cardiovascular risk factors, neurological severity (i.e., muscle strength and NIHSS) at the time of admission and discharge, laboratory findings, brain images, and electrophysiologic results of the subjects.

### Modified Raynaud scan

**Figure 1:**
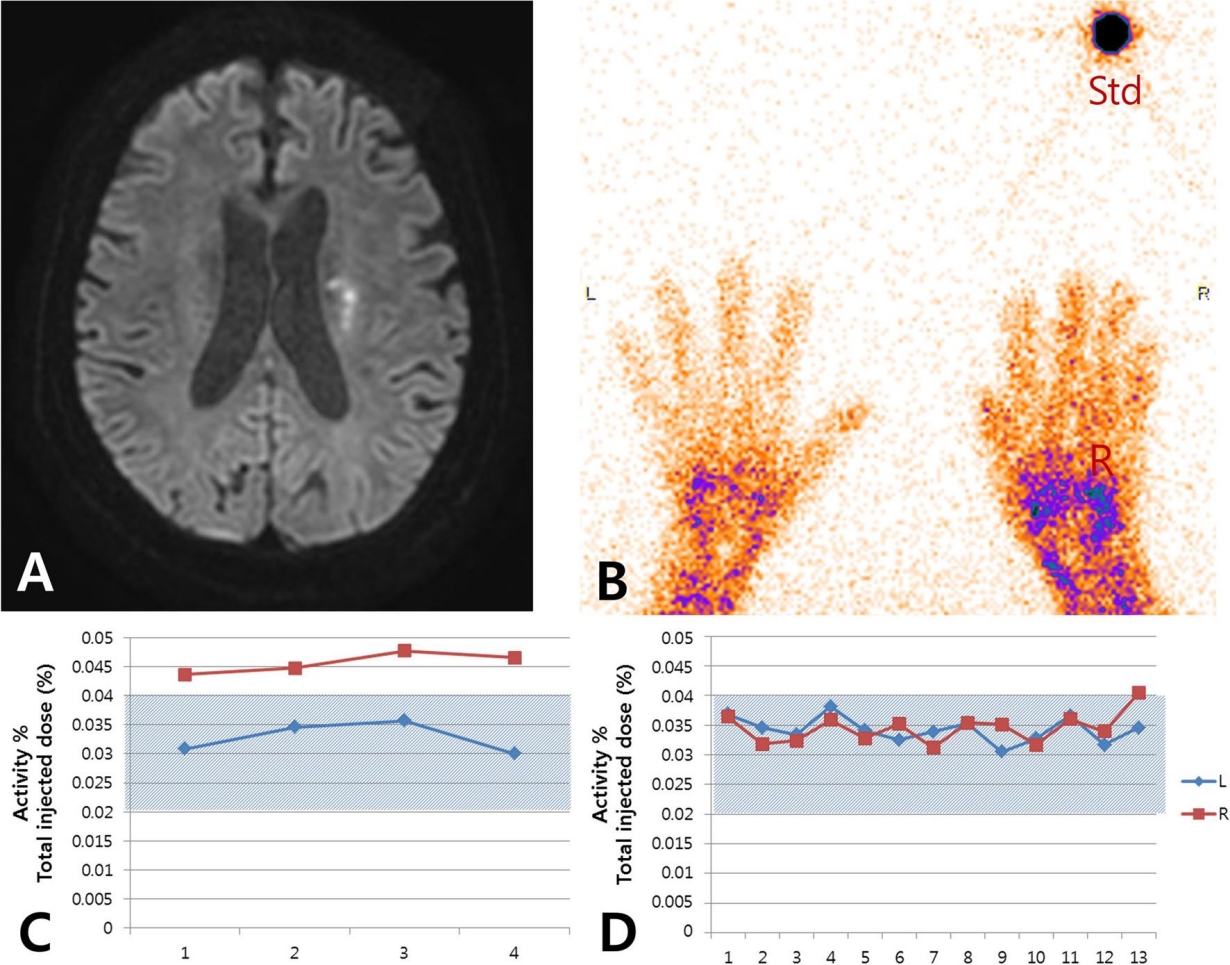
Modified Raynaud scan of preserved vasoreactivity case. (**A**) High signal intensity in left periventricular white matter on the diffusion-weighted image. (**B**) Scan for both hands and standard source. (**C**) Congested right fingertips in resting state. Activity % of the total injected dose means the percentage of total blood. (**D**) Preserved vasoreactivity under thermal stress. The amount of blood in the right hand became equal to the amount in the left hand, and the pattern was stable during the scan. The shaded area in (**C**,**D**) represents the reference range obtained from 10 normal volunteers. R: right; L: left; Std: standard.

**Figure 2:**
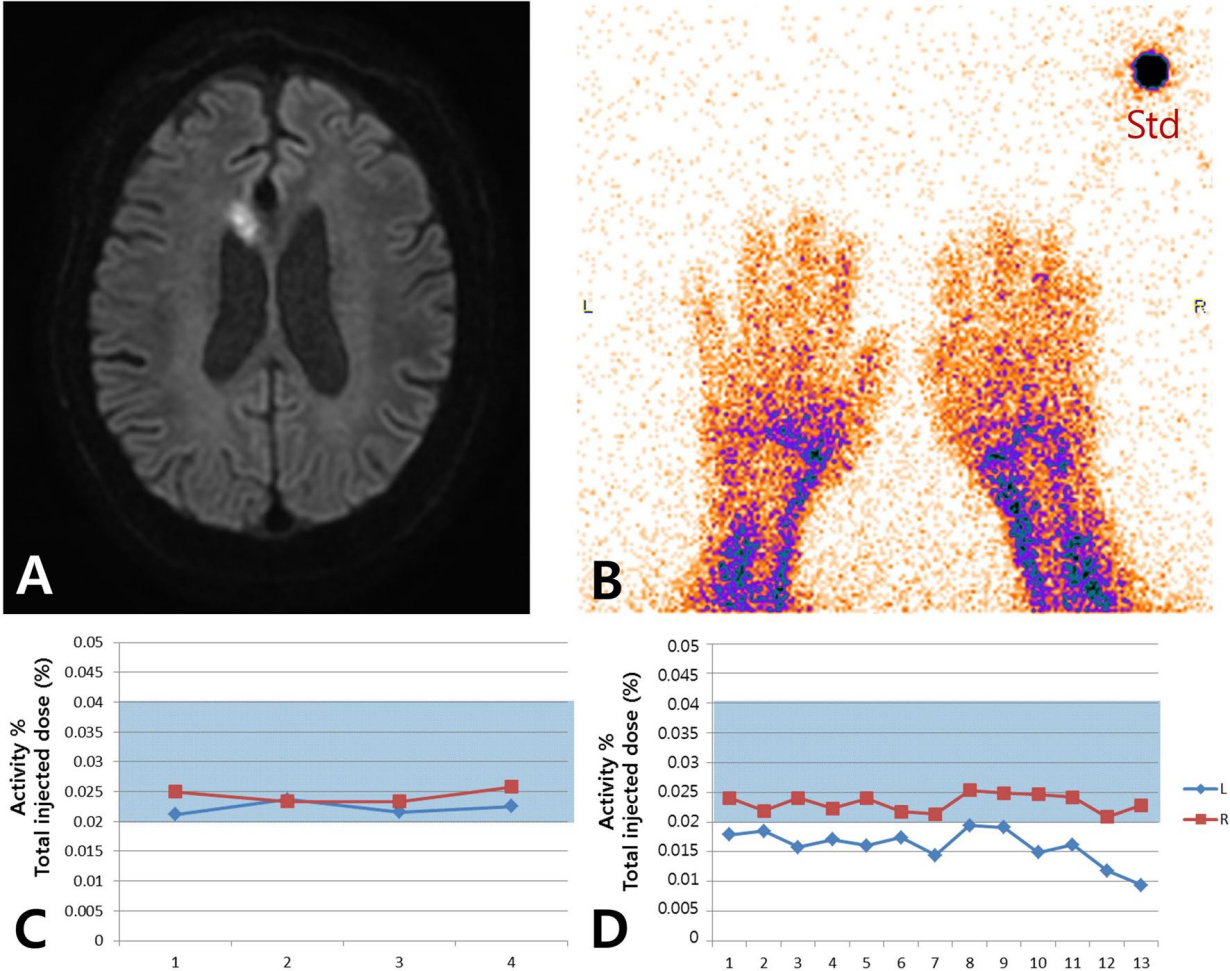
Modified Raynaud scan of impaired vasoreactivity case. (**A**) High signal intensity in right anterior cerebral artery territory on a diffusion-weighted image. (**B**) Scan for both hands and standard source. (**C**) Same blood amount in both hands. Activity % of the total injected dose means the percentage of total blood. (**D**) Impaired vasoreactivity in the left hand under thermal stress. The amount of blood in the right hand was stable during the scan. The shaded area in Fig. 1 (**C**,**D**) represents the reference range obtained from 10 normal volunteers. R: right; L: left; Std: standard.

Red blood cell scintigraphy, an invasive test procedure used in nuclear medicine, is designed for the direct and quantitative assessment of blood amount and blood flow using Tc-99 m labeled RBCs in the blood. First, the pyrophosphates were injected intravenously. Approximately 30 min later, 5 cc of each patient's blood was collected in a 10-cc syringe containing 0.8 mL citrate–phosphate-dextrose-adenine (CPDA) and 15 mCi of 99mTcO4, followed by incubation at room temperature for approximately 10 min. After 5 to 10 min after the injection of the labeled blood, images at rest were acquired using a gamma camera for 2 min. After the rest image was acquired, the patient placed his or her hands on the thermal stress device and stress images were acquired using the same camera for 390 s. During the stress image acquisition, the device was operated using the same treatment cycle (15 °C to 41 °C, 390 s). The gamma camera was set to obtain images at a rate of 1 frame per 3 s for a total of 40 resting images and 130 stress images. One set of rest images and three sets of stress images were obtained before and after treatment. PMOD software (version 3.7, PMOD Technologies LLC, Zurich, Switzerland) was used to analyze the image data and the count of each ROI in this study. The count per time of each region was used to calculate the amount of blood, as compared to the standard source using Excel 2010 software (Microsoft Corporation, Redmond, WA, USA). The images were interpreted using a quantitative graph, and more than 50% of blood flow change during stress was categorized as impaired vasoreactivity. The reference values were set by 10 volunteers. Representative cases of preserved and impaired vasoreactivity determined by the modified Raynaud scan are presented in Figs. 1 and 2.

### Selection of stroke patients

Patients who had neurological deficits within 7 days from the date of admission underwent brain MRI and MR angiography, involving the carotid arteries. An acute ischemic lesion was confirmed using a high signal intensity in a diffusion-weighted image (DWI) map and low signal intensity in a corresponding area in an apparent diffusion coefficient (ADC) map. The involvement of the corticospinal tract in the lesion that presented with hemiplegia was investigated. The corticospinal tract includes the following lesions based on anatomical structure: primary motor cortex, corona radiate, centrum semiovale, internal capsule, cerebral peduncle, and pyramid. Only patients showing acute ischemic lesions with neuroanatomically related motor weakness were included in the final analysis.

### Statistical analysis

Student's *t*-tests were used to analyze continuous variables, and Pearson's chi-squared tests were used to analyze categorical variables. A *p*-value of ≤ 0.05 was considered statistically significant. Binary logistic regression was used to evaluate the factors influencing the results of the modified Raynaud scan. It included *p*-values, adjusted odds ratios (aOR), and 95% confidence intervals (CI). The statistical tests were performed using SPSS 25.0 (Windows, IBM Corp., Armonk, NY).

## Results

In the resting state, 45 patients (65.2% of a total of 69 patients) showed similar blood flow in both hands. In 10 patients (14.5%), the hemiplegic side showed lower blood flow than the unaffected side. A total of 14 patients (20.3%) had higher blood flow on the hemiplegic side than on the contralateral side. There was no statistical difference between the two groups classified according to the reactivity under temperature stress (Supplementary Table 1).

**Table 1:** Baseline characteristics of study population.

Variables	Raynaud scan ±		*p* value
	Preserved (n = 37)	Impaired (n = 32)	
**Demographics**			
Age (years)	69.1 ± 10.6	62.6 ± 11.8	0.019
Male (%)	25 (67.6)	24 (75.0)	0.497
**Conventional risk factors (%)**			
Hypertension	24 (64.9)	15 (46.9)	0.133
Diabetes mellitus	16 (43.2)	9 (28.1)	0.193
Dyslipidemia	11 (29.7)	8 (25.0)	0.661
Atrial fibrillation	3 (8.1)	5 (15.6)	0.457
Smoking	8 (21.6)	13 (40.6)	0.087
Previous stroke	8 (21.6)	4 (12.5)	0.359
**Neurological Severity**			
Proximal muscle strength	4 [4–4.5]	4 [3.25–4]	0.836
Distal muscle strength	4 [3.5–4.5]	4 [3.25–4.75]	0.633
NIHSS at admission	4 [2–6]	4.5 [2.25–6.75]	0.456
NIHSS at discharge	2 [0.5–3]	2 [1–3.75]	0.636
**Associated symptoms (%)**			
Affected limb edema	24 (64.9)	29 (90.6)	0.020
Abnormal sensation	10 (27.0)	7 (21.9)	0.620

*NIHSS* National Institutes of Health Stroke Scale.

The results of the modified Raynaud scan placed 37 subjects (mean age 69.1 ± 10.6) in the preserved group and 32 subjects (mean age 62.6 ± 11.8) in the impaired group. The participants in the impaired group were significantly younger (*p* = 0.019), and they had a higher rate of affected limb edema (preserved 64.9% vs. impaired 90.6%, *p* = 0.02) and anterior circulation distribution of lesions (preserved 62.2% vs. impaired 84.4%, *p* = 0.039) than those in the preserved group. Conventional risk factors, neurological severity including the degree of weakness, and laboratory results of the two groups were not significantly different (Tables 1, 2).

**Table 2:** Diagnostic features of study population.

Variables	Raynaud scan		*p* value
	Preserved (n = 37)	Impaired (n = 32)	
**Stenosis of artery (%)**			
Intracranial	14 (37.8)	13 (40.6)	0.813
Extracranial	6 (16.2)	8 (25.0)	0.366
**Distribution of lesion (%)**			
Anterior circulation	23 (62.2)	27 (84.4)	0.039
Cortex	7 (18.9)	10 (31.3)	0.236
**Electrophysiologic test (%)**^€^			
QSART	25 (92.6)	14 (77.8)	0.199
SSR	4 (14.8)	3 (16.7)	0.867
**Laboratory findings**			
White blood cell (10^3^/μl)	7.96 ± 2.31	8.06± 2.81	0.868
Hemoglobin (100^3^/μl)	14.1 ± 1.7	14.1 ± 2.04	0.994
Platelet (10^3^/μl)	246.0 ± 57.6	256.8 ± 75.6	0.506
ESR (mm/hr)	15.5 ± 11.1	15.5 ± 13.9	0.989
hs-CRP (mg/L)	5.2 ± 11.2	3.9 ± 10.4	0.622
Total cholesterol (mg/dl)^*^	169.4± 34.5	171.4 ± 32.9	0.810
Triglycerides (mg/dl)	158.3± 100.3	163.3 ± 88.8	0.834
HDL (mg/dl)	41.0 ± 9.5	43.2 ± 12.8	0.433
LDL (mg/dl)	108.6 ± 35.9	106.6 ± 32.5	0.836
HbA1c (%)	6.5 ± 1.4	6.5 ± 1.6	0.885

*QSART* Quantitative sudomotor axon reflex test, *SSR* sympathetic skin response, *ESR* erythrocyte sedimentation rate, *hs-CRP* high sensitivity C-reactive protein, *HDL* high density lipoprotein, *LDL* low density lipoprotein, *HbA1c* hemoglobin A1c.

^*^Lipid profile (Preserved 36, Impaired 30).

^€^Preserved 27, Impaired 18.

Logistic regression analysis was carried out to identify the factors that influenced the results of the modified Raynaud scan (Table 3). The results indicated that limb edema (OR 6.15; CI 1.40–26.97; *p* = 0.016) and anterior circulation (OR 3.68; CI 1.01–13.48; *p* = 0.049) significantly influenced the results.

**Table 3:** Logistic regression analysis according to Raynaud scan results.

Variables	Univariate analysis		Multivariate analysis^*^	
	Crude OR (95% CI)	*p* value	Adjusted OR (95% CI)	*p* value
**Demographics**				
Age (years)	0.95 (0.91–0.99)	0.023	0.96 (0.91–1.01)	0.106
Sex	0.69 (0.24–2.00)	0.498		
**Associated symptoms**				
Affected limb edema	3.29 (1.03–10.51)	0.045	6.15 (1.40–26.97)	0.016
Abnormal sensation	0.76 (0.25–2.29)	0.621		
**Conventional risk factors (%)**				
Hypertension	0.48 (0.18–1.26)	0.135		
Diabetes mellitus	0.51 (0.19–1.41)	0.195		
Dyslipidemia	0.79 (0.27–2.29)	0.661		
Atrial fibrillation	2.10 (0.46–9.58)	0.338		
Smoking	2.48 (0.87–7.11)	0.091	0.995 (0.28–3.50)	0.994
Previous stroke	0.52 (0.14–1.92)	0.324		
**Neurological Severity**				
Proximal muscle strength	0.95 (0.58–1.56)	0.833		
Distal muscle strength	1.12 (0.71–1.77)	0.628		
**Stenosis of artery**				
Intracranial	1.12 (0.43–2.96)	0.813		
Extracranial	1.72 (0.53–5.63))	0.369		
**Distribution of ischemic lesion**				
Anterior circulation	5.24 (1.34–20.54)	0.018	3.68 (1.01–13.48)	0.049
Cortex	1.95 (0.64–5.92)	0.240		
**Laboratory findings**				
White blood cell (10^3^/μl)	1.02 (0.84–1.23)	0.866		
Hemoglobin (100^3^/μl)	1.00 (0.77–1.29)	0.994		
Platelet (10^3^/μl)	1.00 (1.00–1.01)	0.502		
ESR (mm/hr)	1.00 (0.96–1.04)	0.989		
hs-CRP (mg/L)	0.99 (0.94–1.04)	0.622		
Total cholesterol (mg/dl)	1.00 (0.99–1.02)	0.806		
Triglycerides (mg/dl)	1.00 (1.00–1.01)	0.831		
HDL (mg/dl)	1.02 (0.97–1.07)	0.429		
LDL (mg/dl)	1.00 (0.98–1.01)	0.832		
HbA1c (%)	0.98 (0.70–1.35)	0.883		

*OR* odds ratio, *CI* confidence interval, *ESR* erythrocyte sedimentation rate, *hs-CRP* high sensitivity C-reactive protein, *HDL* high density lipoprotein, *LDL* low density lipoprotein, *HbA1c* hemoglobin A1c.

^*^Adjusted for age, smoking, affected limb edema, anterior circulation, and cortex.

## Discussion

In the present study, the modified Raynaud scan was used for patients with acute cerebral infarction showing unilateral weakness to assess the peripheral vasoreactivity under the circumstance of central lesion. Through the study, edematous limb (OR 6.15; CI 1.40–26.97; *p* = 0.016) and lesions in the anterior circulation (OR 3.68; CI 1.01–13.48; *p* = 0.049) significantly influenced peripheral vasoreactivity under temperature stress.

Raynaud's phenomenon (RP) is a form of dysautonomia with a prevalence of approximately 3%–5% of the general population, and it is more common in women^6,7^. Abnormal vasoreactivity of the peripheral arteries due to temperature change is a key clinical manifestation. When exposed to cold temperature, the skin color of the extremities usually undergoes three stages of change due to dysfunctional vasoreactivity. RP is classified into primary and secondary depending on the etiology. Primary RP does not have an established etiology^8^, and it is common in young women under 30 years of age and generally benign. Secondary RP is caused by several secondary causal factors such as vascular disease, autoimmune disease, medication, and habitual situations^1,9^. However, ischemic stroke has not been considered as an etiology of impaired vasoreactivity in the peripheral blood system.

Several methodologies have been developed to precisely measure peripheral blood flow. Infrared thermography is a method that measures the skin temperature of the extremities using infrared rays. It can be used to distinguish patients with from those without dysautonomia and differentiate between vasoreactivity dysfunction subtypes when considering rewarming time after the skin is cooled^9^. Infrared thermography also provides quantitative details by measuring the difference between the temperatures of the fingers and the back of the hand^10^. However, it dose not provide accurate information of depth, and is expensive. Laser speckle contrast imaging, which has gained popularity in recent years, is a method that uses the speckle pattern created after a laser hits the RBCs. The speckle pattern changes because of the RBC movement, and the changes are measured using a camera^11,12^. Laser speckle contrast imaging shows a high correlation with thermography in patients with systemic sclerosis. Additionally, laser Doppler flowmetry (LDF), based on the Doppler effect, is used to evaluate peripheral blood flow^13–15^. However, it cannot accurately reflect overall blood flow at the measurement site because it can only detect blood flow in blood vessels for a restricted diameter.

The conventional Raynaud scan has been used to qualitatively validate peripheral blood supply and microvascular vasoreactivity of the upper and lower extremities. The modified Raynaud scan applied in this study used a repetitive and cyclic cooling-heating production device that was developed for pain release using thermal therapy as a stress tool to acquire dynamic data during scanning. This device delivered temperatures ranging from 15 to 41 °C to the examined palms. It was used to quantify blood flow by calculating absolute values, which were obtained by comparison with a standard radioactivity value and volume source. This study showed differences in patients without visible dysautonomia, which indicates that the modified Raynaud scan may be highly sensitive for evaluating vasoreactivity. Additionally, it is suitable for evaluating vasoreactivity under stress, because it is possible to obtain images under varying thermal stress for up to 48 h with one RBC labeling.

Anatomically, the peripheral nervous system branches out from the sympathetic nervous system at the T1-L2 level. When exposed to cold, norepinephrine is secreted by activating the sympathetic nervous system, which innervates the peripheral artery. When the alpha-1 adrenergic receptor distributed in blood vessels binds to the secreted norepinephrine, it reduces blood flow by increasing vasomotor tone (vasoconstriction). At the level above the spinal cord, the hypothalamus and anterior cingulate cortex (ACC) are involved in peripheral vasoconstriction. Peripheral temperature signals are integrated into the preoptic area of the hypothalamus. If the body temperature is lower than the set-point, the sympathetic preganglionic neuron in the intermediolateral nucleus is stimulated, which eventually causes peripheral vasoconstriction^16^. Moreover, it was reported in a rat model that perfusion of the peripheral artery changed following the electrical stimulation of the ACC, and the lateral hypothalamus was involved in the process^2^. Theoretically, the brain parenchyma may also be associated with vasoconstriction, but this has not yet been confirmed. Our results suggest that the anterior circulation, including the cerebral hemisphere, is associated with vasoreactivity. The results also suggest that the functionally highest level of the central neuvous system is associated with the regulation of the autonomic nervous system.

Peripheral edema often occurs in limbs with weakness after a stroke^17^. It is associated with the venous return caused by immobilization of the paralyzed limb and functional disorders of the lymph system^18^. Chronic peripheral edema is also associated with pain and fibrotic tissue^19^. Our results showed that the modified Raynaud scan was more likely to detect an abnormality in the edema patient group. Additional analysis showed that an abnormality of the modified Raynaud scan was significantly related to the occurrence of edema (data not shown). However, the weakness of the limbs did not influence the occurrence of edema, indicating that peripheral edema cannot be explained by immobilization alone, and abnormal vasoreactivity may be related to it. A causal relationship between peripheral edema and the modified Raynaud scan outcome is difficult to establish. However, considering the methodology of the modified Raynaud scan, it is unlikely that edema affected the modified Raynaud scan results. In other words, the results of this study demonstrate that the impaired group, based on the results of the modified Raynaud scan, is likely to develop peripheral edema.

This study has several limitations. First, the modified Raynaud scan findings before the onset of a stroke were not obtained. We cannot rule out the possibility that patients with cerebral infarction would have shown abnormal modified Raynaud scan results before the onset of stroke. This should be verified through prospective cohort studies. Second, we could not explain the exact pathophysiological relationship between stroke and impaired peripheral vasoreactivity. The results of this study have shown that the two are related; therefore, further research on related mechanisms or more diverse clinical associations with other findings are needed. Lastly, patients with severe weakness or aphasia were excluded from this study. This exclusion may have caused a selection bias.

In conclusion, brain hemisphere lesions can affect peripheral vasoreactivity, and they are associated with peripheral edema via abnormal vasoreactivity. These results may help medical staff to plan for rehabilitation in stroke patients with hemiplegia. It is necessary to compare the results of the modified Raynaud scan with the results of other methods and measure reference data in the future.

## Supplementary Information

Supplementary Information.
