# Author Correction: Peripheral vasoreactivity in acute ischemic stroke with hemiplegia

**DOI:** 10.1038/s41598-021-98196-1

**Published:** 2021-09-15

**Authors:** Su Jeong Wang, Chan‑Hyuk Lee, Hyun Goo Kang, Ko Woon Kim, Minjoo Kim, Hwan‑Jeong Jeong, Byoung‑Soo Shin

**Affiliations:** 1 Department of Neurology, Jeonbuk National University Medical School and Hospital, 20 Geonji‑ro, Deokjin‑gu, Jeonju, 54907 Republic of Korea; 2 Research Institute of Clinical Medicine of Jeonbuk National University-Biomedical Research Institute of Jeonbuk National University Hospital, Jeonju, Republic of Korea; 3 Department of Nuclear Medicine, Jeonbuk National University Medical School and Hospital, 20 Geonji‑ro, Deokjin‑gu, Jeonju, 54907 Republic of Korea

Correction to: *Scientific Reports* 10.1038/s41598-021-88050-9, published online 20 April 2021

The original version of this Article contained errors in Tables 1, 2 and 3 where previous versions of the tables were published.

The original Tables 1, 2 and 3 and accompanying legends appear below.

**Table 1:** Baseline characteristics of study population.

Variables	Raynaud scan		*p* value
	Preserved (n = 34)	Impaired (n = 35)	
**Demographics**			
Age (years)	69.3 ± 10.2	63.0 ± 12.1	0.022
Male (%)	23 (67.6)	26 (74.3)	0.543
**Conventional risk factors (%)**			
Hypertension	22 (64.7)	17 (48.6)	0.176
Diabetes mellitus	14 (41.2)	11 (31.4)	0.400
Dyslipidemia	10 (29.4)	9 (25.7)	0.731
Atrial fibrillation	2 (5.9)	6 (17.1)	0.259
Smoking	6 (17.6)	15 (42.9)	0.023
Previous stroke	7 (20.6)	5 (14.3)	0.490
**Neurological Severity**			
Proximal muscle strength	4 [4–5]	4 [3.25–4]	0.390
Distal muscle strength	4 [3.75–5]	4 [3.25–4.75]	0.924
NIHSS at admission	4.5 [2–6]	4 [2–6.75]	0.325
NIHSS at discharge	2 [0–3]	2 [1–3.75]	0.308
**Associated symptoms (%)**			
Affected limb edema	21 (61.8)	32 (91.4)	0.004
Abnormal sensation	9 (26.5)	8 (22.9)	0.728

*NIHSS* National Institutes of Health Stroke Scale.

**Table 2:** Diagnostic features of study population.

Variables	Raynaud scan		*p* value
	Preserved (n = 34)	Impaired (n = 35)	
**Stenosis of artery (%)**			
Intracranial	12 (35.3)	15 (42.9)	0.520
Extracranial	6 (17.6)	8 (22.9)	0.591
**Distribution of lesion (%)**			
Anterior circulation	20 (58.8)	30 (85.7)	0.012
Cortex	5 (14.7)	12 (34.3)	0.059
**Electrophysiologic test (%)** ^b^			
QSART	22 (91.7)	17 (81.0)	0.396
SSR	4 (16.7)	3 (14.3)	1.000
**Laboratory findings**			
White blood cell (10^3^/µl)	7.75 ± 2.28	8.25 ± 2.78	0.424
Hemoglobin (100^3^/µl)	14.08 ± 1.77	14.09 ± 1.96	0.979
Platelet (10^3^/µl)	248.65 ± 58.44	253.26 ± 73.88	0.775
ESR (mm/hr)	14.38 ± 10.79	16.63 ± 13.82	0.455
hs-CRP (mg/L)	5.22 ± 11.70	3.97 ± 9.95	0.631
Total cholesterol (mg/dl)^a^	169.5 ± 34.8	171.2 ± 32.7	0.833
Triglycerides (mg/dl)	159.8 ± 103.0	161.3 ± 87.0	0.948
HDL (mg/dl)	40.6 ± 8.6	43.3 ± 13.1	0.326
LDL (mg/dl)	109.0 ± 36.5	106.6 ± 32.1	0.721
HbA1c (%)	6.5 ± 1.4	6.5 ± 1.6	0.833

*QSART* Quantitative sudomotor axon reflex test, *SSR* sympathetic skin response, *ESR* erythrocyte sedimentation rate, *hs-CRP* high sensitivity C-reactive protein, *HDL* high density lipoprotein, *LDL* low density lipoprotein, *HbA1c* hemoglobin A1c.

^a^Lipid profile (Preserved 33, Impaired 34).

^b^Preserved 24, Impaired 22.

**Table 3:** Logistic regression analysis according to Raynaud scan results.

Variables	Univariate analysis		Multivariate analysis^a^	
	Crude OR (95% CI)	*p* value	Adjusted OR (95% CI)	*p* value
**Demographics**				
Age (years)	0.951 (0.909–0.994)	0.026		
Sex	0.724 (0.255–2.057)	0.544		
**Associated symptoms**				
Affected limb edema	6.603 (1.677–26.005)	0.007	15.364 (2.774–85.087)	0.002
Abnormal sensation	0.823 (0.275–2.465)	0.728		
**Conventional risk factors (%)**				
Hypertension	0.515 (0.196–1.354)	0.179		
Diabetes mellitus	0.655 (0.244–1.758)	0.401		
Dyslipidemia	0.831 (0.288–2.392)	0.731		
Atrial fibrillation	3.310 (0.619–17.715)	0.162		
Smoking	3.500 (1.157–10.589)	0.027		
Previous stroke	0.643 (0.182–2.266)	0.492		
**Neurological Severity**				
Proximal muscle strength	0.798 (0.478–1.330)	0.386		
Distal muscle strength	0.978 (0.622–1.537)	0.923		
**Stenosis of artery**				
Intracranial	1.375 (0.521–3.631)	0.520		
Extracranial	1.383 (0.424–4.514)	0.591		
**Distribution of ischemic lesion**				
Anterior circulation	4.200 (1.307–13.497)	0.016	4.540 (1.257–16.397)	0.021
Cortex	3.026 (0.932–9.829)	0.065	4.885 (0.893–26.723)	0.067
**Laboratory findings**				
White blood cell (10^3^/µl)	1.082 (0.894–1.309)	0.419		
Hemoglobin (100^3^/µl)	1.004 (0.777–1.296)	0.978		
Platelet (10^3^/µl)	1.001 (0.994–1.008)	0.771		
ESR (mm/hr)	1.015 (0.976–1.056)	0.451		
hs-CRP (mg/L)	0.989 (0.944–1.035)	0.630		
Total cholesterol (mg/dl)	1.002 (0.987–1.016)	0.830		
Triglycerides (mg/dl)	1.000 (0.995–1.005)	0.947		
HDL (mg/dl)	1.023 (0.978–1.071)	0.325		
LDL (mg/dl)	0.998 (0.984–1.012)	0.774		
HbA1c (%)	1.036 (0.748–1.435)	0.830		

*OR* odds ratio, *CI* confidence interval, *ESR* erythrocyte sedimentation rate,* hs-CRP* high sensitivity C-reactive protein,* HDL* high density lipoprotein, *LDL* low density lipoprotein,* HbA1c* hemoglobin A1c.

^a^Adjusted for age, smoking, affected limb edema, anterior circulation, and cortex.

The original Article has been corrected.

