# Supplementary material for: Peripheral vasoreactivity in acute ischemic stroke with hemiplegia
Source: Sci Rep. 2021 Apr 20;11:8531. doi: 10.1038/s41598-021-88050-9 (PMC8058338; doi:10.1038/s41598-021-88050-9)
Supplement: Supplementary file 1 — Supplementary Information. [file 41598_2021_88050_MOESM1_ESM.docx]

Supplementary table

**Peripheral vasoreactivity in acute ischemic stroke with hemiplegia**

Su Jung Wang MD^1^, Chan-Hyuk Lee MD^1^, Hyun Goo Kang MD, PhD^1,2^ , Ko Woon Kim MD, PhD ^1,2^; Minjoo Kim PhD^3^, Hwan-Jeong Jeong MD, PhD^3^*; Byoung-Soo Shin MD, PhD^1,2^*

^1^ Department of Neurology, Jeonbuk National University Medical School and Hospital, Jeonju, South Korea

^2^ Research Institute of Clinical Medicine of Jeonbuk National University-Biomedical Research Institute of Jeonbuk National University Hospital, Jeonju, South Korea

^3^Department of Nuclear Medicine, Jeonbuk National University Medical School and Hospital, Jeonju, South Korea

| **Supplementary table 1. Blood flow compared to an unaffected limb in resting state** | | | |
| --- | --- | --- | --- |
| Blood flow pattern | Stress-induced state | | *p* value |
|  | Preserved (n=37) | Impaired (n=32) |  |
| Similar | 22 | 23 |  |
| Decreased | 7 | 3 | *0.460* |
| Increased | 8 | 6 |  |
